# Immune Microenvironment and Genetics in Malignant Pleural Mesothelioma

**DOI:** 10.3389/fonc.2021.684025

**Published:** 2021-06-11

**Authors:** Benjamin Wadowski, Raphael Bueno, Assunta De Rienzo

**Affiliations:** Thoracic Surgery Oncology Laboratory and the International Mesothelioma Program, Division of Thoracic and Cardiovascular Surgery, Brigham and Women’s Hospital and Harvard Medical School, Boston, MA, United States

**Keywords:** mesothelioma, genomics, transcriptomics, immune, checkpoint, microenvironment

## Abstract

Malignant pleural mesothelioma (MPM) is a rare and aggressive malignancy with limited therapeutic options beyond surgery and cytotoxic chemotherapy. The success of immune checkpoint inhibition has been found to correlate with expression of immune-related genes such as *CD274* (PD-L1) in lung and other solid cancers. However, only a small subset of MPM patients respond to checkpoint inhibition, and this response has been varied and unpredictable across several clinical trials. Recent advances in next-generation sequencing (NGS) technology have improved our understanding of the molecular features of MPM, also with respect to its genetic signature and how this impacts the immune microenvironment. This article will review current evidence surrounding the interplay between MPM genetics, including epigenetics and transcriptomics, and the immune response.

## Introduction

Malignant pleural mesothelioma (MPM) is a rare and aggressive tumor of the pleural cavity. It affects approximately 3,000 new patients per year in the United States, and median survival following diagnosis ranges from 7 to 13 months (1, 2). First-line treatment consists of cytotoxic chemotherapy either in the neoadjuvant or adjuvant setting (3). Given the success of immune checkpoint inhibition in other solid tumors, the use of these agents is being investigated in MPM. Unfortunately, few MPM patients respond to current checkpoint inhibitor regimens (4, 5) and reliable predictive biomarkers for response are lacking (6). A deeper understanding of the immune microenvironment in MPM is required to improve the way immunotherapy is applied in these patients. In this review, we explore the interplay between the complex molecular features of MPM and the immune response.

## The Immune Microenvironment in MPM

### Tumor Development

MPM is believed to arise in the context of chronic inflammation (7). It is associated with asbestos exposure in 70-90% of cases (8). Over decades, asbestos or other mineral fibers can cause both direct cytotoxicity and genotoxicity, generate free radicals, and lead to chronic inflammation through cytokine dysregulation (9, 10). This in turn results in immune activation, propagating the inflammatory environment and contributing to epigenetic and genetic alterations in mesothelial cells, and eventual malignant transformation (11). MPM may also arise in the absence of prolonged inflammation, particularly in young female patients or those with germline variants in genes such as *BRCA1* associated protein 1 (*BAP1*) and BLM RecQ Like Helicase (*BLM*) (12, 13). However, MPM tumorigenesis under these circumstances is rare and remains incompletely understood.

Despite evidence for the typical role of inflammation in MPM oncogenesis, tumor survival requires an element of immune evasion or immunosuppression: the tumor microenvironment is believed to be highly immunosuppressive in MPM (11). Consequently, the composition of the immune cell infiltrate, including macrophage phenotypes and lymphocyte subpopulations, has been investigated in several studies.

### The Immunosuppressive Phenotype

The interactions between myeloid cells, particularly tumor-associated monocytes/macrophages (TAMs), and lymphoid cells regulate the local antitumor immune response. In particular, different macrophage phenotypes can shape the immune microenvironment in divergent ways: classically activated M1 macrophages promote T cell proliferation and antitumor activity, while alternatively activated M2 macrophages exert immunosuppressive effects *via* cytokines such as IL-6 and IL-10 (14). Prevalence, function, and prognostic implications of these cell types have been extensively investigated in MPM (11).

To study the myeloid infiltrate in MPM tumors, Burt and collaborators (15) performed immunohistochemistry (IHC) for CD68, a macrophage surface marker, in 52 MPM tumors. They found that macrophages comprise a significant (27% ± 9%) proportion of tumor area on average. The same group found that the numbers of preoperative circulating monocytes and total white blood cells (WBC) were higher in non-epithelial compared with epithelioid MPM. In addition, higher preoperative monocyte counts were correlated with overall shorter survival in all patients regardless of histology (HR 3.98 [2.64-5.93] p<0.001). Successively, Ujiie and collaborators (16) demonstrated that beyond macrophage prevalence, the proportion of M2 macrophages specifically influences prognosis. Within the tumor, monocytes differentiate into immunosuppressive macrophages *via* the CSF1R pathway in response to M-CSF (17, 18) and potentially IL-34 secretion by tumor cells (19). Furthermore, IHC analysis for a panel of immune-related markers was performed on 395 MPM tumors across the histologic spectrum. Shorter survival was associated with increased CD163/CD8 (N=22) and CD163/CD20 (N=48) ratios, which are indicative of an M2 predominance. A separate IHC-based analysis in epithelioid tumors alone showed a similar decrease in survival with higher CD163/CD68 ratio (Pearson r −0.72, p<0.05), demonstrating the deleterious effect of M2 polarization (14). Using bulk RNA-seq data, Bueno and collaborators (20) found that the M2 macrophage to T cell ratio based on the expression levels of 41 genes was predictive of reduced overall survival. In addition, the expression levels of the 22 genes associated with M2 macrophage phenotype were estimated to be higher in sarcomatoid tumors confirming previous observations of higher number of macrophages in non-epithelioid tumors.

To characterize the lymphoid infiltrate in MPM, Awad and collaborators (21) utilized a novel method for comprehensive immune profiling using flow cytometry in 43 MPM tumors annotated with programmed death ligand 1 (PD-L1) IHC status. PD-L1–positive and non-epithelioid tumors showed a significantly greater proportion of infiltrating T cells than PD-L1–negative and epithelioid tumors (21). In addition, PD-L1–positive tumors exhibited considerable immunophenotypic variability across samples, with a higher proportion of CD8+ memory T cells (p = 0.007), higher CD8+ effector memory T cells (p = 0.03), and a lower proportion of CD8+ effector T cells (p = 0.001) than the PD-L1 negative tumors. Moreover, PD-L1 expression was shown to be associated with increased CD8 T cell proliferation (based on Ki67+ status) and with increased proportion of Treg infiltration compared to PD-L1 negative tumors (21). This study suggested that the immunophenotypic variability observed across PD-L1 samples may be responsible for the minority of PD-L1–positive mesotheliomas likely to respond to pembrolizumab. Another study by Combaz-Lair and colleagues (22) examined the association between PD-L1 staining, TLR3 expression and immune infiltration by IHC in 58 MPM FFPE specimens. The authors demonstrated, using two different antibodies, that overall PD-L1 expression was increased in sarcomatoid tumors compared with other histologic types. A correlation between PD-L1 expression on infiltrating lymphocytes and PD-L1 expression on tumor cells was also found (p ≤ 0.001 for both antibody clones). In addition, a correlation between PD-L1 expression on lymphocytes and CD3 and CD8 expression was found, but there was no association between PD-L1 expression and immune infiltrate density identified in this study. Increased PD-L1 expression was associated with reduced unadjusted survival for one clone (SP142, log-rank p=0.016) but not the other (E1L3L, log-rank = 0.022) (22). A different study characterized 93 treated and 65 chemo-naïve MPM cases by tumor microenvironment (TME) and PD-L1 status, respectively (23). Non-epithelioid tumors showed higher cytotoxic T cell infiltration, higher macrophage infiltration, and lower CD4+ T cell levels than epithelioid tumors; they also showed higher levels of tumor PD-L1 expression. The authors also demonstrated an association of these features with aggressive histopathological characteristics including necrosis and tumor grade. In another IHC analysis of 88 MPM tumors, PD-L1 was expressed regardless of the MPM histologic subtype, but both PD-L1 positive tumors and sarcomatoid MPM showed an increase of stromal CD4+ and CD19+ lymphocytes. In contrast, epithelioid tumors were associated with a higher proportion of CD8+ cells (24).

In addition to their effect on the TIL composition, the relative composition of the lymphoid infiltrate itself was found to be an independent predictor of outcomes. The balance between CD4 and CD8 T cells is key in this respect (24, 25). Fusco and collaborators (24) analyzed 88 MPM tumors. In this study, CD4+ cells correlated with improved prognosis (HR 0.48 [0.24-0.96] p=0.036), while CD8 infiltration correlated with poor prognosis (for low CD8, HR 0.44 [0.27-0.72] p=0.0012). Consequently, a high stromal CD4/CD8 ratio was found to be an independent predictor of longer survival in a multivariate model accounting for histology and PD-L1 status (24). Moreover, associations between tumor infiltrating lymphocytes (TIL) and survival were observed to change in presence of systemic therapy. A separate analysis of 32 MPM specimens, resected post-neoadjuvant chemotherapy, demonstrated that patients with high levels of CD8+ tumor-infiltrating lymphocytes by IHC had longer survival compared with those with low levels (3-year survival: 83% vs. 28%; p = 0.06) (26).

In summary, lymphoid and myeloid cells comprise a significant portion of the MPM microenvironment. Non-epithelioid histologic subtypes often exhibit an immunosuppressive phenotype, which correlates with shorter survival. However, there remains significant variability even among histologically similar tumors and further work is needed to explore how this variability may influence treatment response.

### Genetic and Epigenetic Effects on the Immune Response

The genetic intratumor heterogeneity is crucial for cancer invasion, proliferation and resistance to therapy and is closely related to the TME (27). The epigenetic and genetic landscape of MPM is characterized by frequent chromosomal losses and a relatively low number of somatic mutations compared to other solid tumors (20). Potential relationships between genome-level features and immune microenvironment in MPM have been investigated including associations with epigenetic, structural/chromosomal, and gene-specific alterations.

Epigenetic modifications determine changes in gene expression without altering the DNA sequence. Epigenetic changes have been associated with tumor progression and poor outcomes in a diverse array of tumors (28–30), and in MPM have been linked to alterations of the tumor-infiltrating immune cells. Epigenome-wide association analyses of a cohort of 159 asbestos-exposed patients with MPM identified the methylation of the single-CpG marker, cg03546163, located in the 5’ untranslated region of the *FKBP5* gene, as associated to survival (31). This marker showed better performance compared with the traditional inflammation scores, lymphocyte-to-monocyte ratio (32), generally used as a prognostic biomarker in MPM.

A similar study focused on immune system-related genes was conducted comparing 163 MPM patients with 137 healthy controls. Several signatures were identified including significant differential methylation of the CpG regions of *LIME1* (involved in lymphocyte signaling), *CXCR6* (associated with T cell localization), *TOLLIP* (related with IL-1 receptor trafficking), and *TNFAIP6* (involved in inflammation) (33). These data supported the hypothesis that changes in the DNA methylation and in the TME may be associated with asbestos exposure.

Chromosomal instability may result in gross karyotypic alterations. It has been related to cancer immunogenicity (27), and is a common feature of MPM (34, 35). Genome-wide copy number analysis performed in 113 MPM tumors with IHC data for PD-L1, CD4, CD8, and FOXP3 was used to compute the percent genome aberration (PGA) for each sample as total count of base pairs involved in copy number gains or losses divided by the total length of the genome in base pairs (36). Epithelioid tumors showed a significantly higher PGA than non-epithelioid tumors, but PGA did not correlate with PD-L1 status. Samples with lower PGA had significantly higher CD4+ and CD8+ T-cell infiltration indicating that chromosomal instability may be associated with immune infiltration. Chromosomal rearrangements have also been shown to have immunologic implications through expression of neoantigens in MPM (37). Mansfield and collaborators (38) performed mate-pair sequencing, RNA-seq, T cell receptor (TCR)-seq, and major histocompatibility complex (MHC) peptide binding assays to assess structural variants of chromosomes and predict neoantigens using 28 specimens from treatment-naïve MPMs. They identified 1535 chromosomal rearrangements, of which 637 (41.5%) resulted in novel gene fusions, leading to 179 potential novel amino acid sequences potentially drive the expression of neoantigens. In addition, the increase in predicted neoantigens was correlated with clonal expansion of tumor-infiltrating T cells. Spatial heterogeneity in MPM has shown to affect TIL clonality in the context of neoantigenicity. In a study of 6 MPM tumors sampled at three distinct anatomic sites each, increasing neoantigen load correlated with oligoclonal TIL expansion, as well as increased cytotoxic T cell activity. In addition, heterogeneous mutation patterns across sites with associated differences in immune microenvironment signatures were identified (39). A multi-region, longitudinal whole exome and T-cell receptor sequencing analysis was conducted on 69 specimens from nine MPM tumors before and after dasatinib treatment. It was found that mutation profile among sites was relatively homogeneous (>80% concordance), but T-cell clonality varied widely particularly after treatment (40).

Growing data suggest that mutations in specific genes influence the immune response (41). *BAP1* is one of the most frequently mutated genes in MPM (20), and the impact of *BAP1* mutations on the immune system has been investigated. An analysis of 43 MPM tumors found no significant differences in the immune cell infiltration between *BAP1* mutant versus wild-type MPM (21). In peritoneal mesothelioma, a multi-omic analysis of 19 tumors identified an association between inflammatory TME and haploinsufficiency of *BAP1*. Specifically, *BAP1* deleted tumors were found to have strong cytokine signaling and upregulation of the innate immune response based on gene set enrichment analysis. In contrast, tumors displaying intact *BAP1* had upregulation of adaptive immunity and MHC-I/II antigen presentation (42). Furthermore, tumors with deleted *BAP1* showed a lower proportion of plasma cells, natural killer (NK) cells, and B cells but higher mast cell and T cell infiltration, as well as higher expression of genes with a known role in immune checkpoint modulation *(PD1*, *PD-L1 CD80*, *CTLA4*, *LAG3*, and *ICOS)*, compared with tumor with wild-type BAP1. This finding was not supported by the TCGA analysis in pleural mesothelioma (42).

Single gene mutations may also influence the antitumor response through neoantigen formation and modulation of immune and inflammatory signaling pathways. Bueno and collaborators (20) analyzed somatic alterations in 98 MPMs with paired exome and RNA-seq data and found that 59% of 1,493 mutations resulted in MHC class I-binding peptides. Further, the more frequently mutated genes *BAP1*, *NF2*, and *TP53* each resulted in multiple predicted neoantigens (20). *CDKN2A* is another frequently mutated gene in MPM (20), and is located <1Mb from several interferon genes raising the possibility that prognostically significant loss of *IFN* gene expression may reflect a “passive hitchhiking event” associated with *CDKN2A* deletion in cancer (43). Type I interferon (IFN-I) signaling is known to play a role in antitumor immunity (44). An integrative analysis of the association between *IFN* gene alterations, *CDKN2A* loss, and *in vitro* oncolytic virus sensitivity was performed. A deletion of *IFNB1* was identified in 17/57 (30%) MPM short-term cell lines with homozygous deletion of *CDKN2A*, and *CDKN2A* loss in 17/18 (94%) established cell lines with homozygous deletion *IFNB1*. Utilizing the TCGA database of 87 patients with MPM, homozygous deletions of *IFNA2* and *IFNB1* were found in 18.4% and 9.2% of patients respectively. Furthermore, homozygous deletion of IFN-I genes resulted in more frequent sensitivity of MPM cell lines to oncolytic virus therapy (45). While a link between IFN signaling and immunotherapy response has not been established in MPM, it has been observed in other cancers (43).

### Heterogeneity of Gene Expression Associated With Immune Checkpoints

Checkpoint molecules such as programmed-death 1 (PD-1) and cytotoxic T lymphocyte associated antigen 4 (CTLA4) have been recognized as key regulators of oncologic immune evasion through their role in immunosuppression (46). Protein expression of checkpoint molecules by IHC such as PD-L1 are often used as prognostic biomarkers to guide response to checkpoint inhibition in several solid cancers (47). However, in MPM, similar analyses have shown inconsistent results (6, 48, 49). In contrast, characterization of the immune microenvironment in relation to checkpoint pathways using high-throughput methodologies has led to prognostic insights both in MPM and other solid tumors (50, 51).

Unsupervised analysis of RNA-seq data from 284 MPMs identified a continuum of molecular profiles which correlate with prognosis (50). The majority of variation was found to be related to immune checkpoint and angiogenic pathways. Two profiles were identified to be associated with poor prognosis: one immunologically active characterized by high lymphocyte infiltration and high immune checkpoint expression, and one less activated profile with low lymphocyte infiltration. Both were characterized by high expression of pro-angiogenic genes. In contrast, a “VEGFR2+/VISTA+” profile was associated with better prognosis, despite having also highly angiogenic features. RNA expression of *VISTA*, a negative checkpoint regulator, was found to be highly expressed in epithelioid MPM in a separate well-annotated cohort of 74 untreated MPM specimens (52).

Blum and collaborators (51) performed an extensive multi-omic analysis using several public MPM transcriptomic datasets. Two signatures (E/S scores) were identified to discriminate epithelioid-like and sarcomatoid-like tumors within a continuum or “histo-molecular gradient” of MPM samples with epithelioid and sarcomatoid tumors at the two extremes. They found that expression of most immune checkpoints correlated with increased S-score, including *TNFSF4* and its receptor *TNFRSF4*, *CD80*, and *PD-L2*, as well as *CD274* and *CTLA4*. A positive association was found between the S-score and *IDO1*, an immune modulator. In contrast, the E-score was associated with *TNFSF14* and *VISTA* expression. Tumors with higher S-score were associated with increased T cell and monocyte infiltration, while E-score was associated with increased NK cell infiltration (51).

## Discussion

Tumor-immune interactions are complex regardless of the tumor type. As immune checkpoint inhibition gains importance in the treatment of solid tumors including MPM, greater emphasis is being placed on immune characterization and identification of predictive biomarkers for treatment response. Several studies have tried to characterize the immune TME of MPM and linked it to clinical and genetic features.

In general, MPM displays an immunosuppressive microenvironment mediated by M2 macrophages and characterized by high lymphocyte infiltration (**Table 1**) (15, 25). This immunosuppressive phenotype is more common in non-epithelioid MPM (20) and as a result the degree of overall macrophage infiltration has been associated with shorter survival in non-epithelioid MPM alone (14, 15). Across all histologic subtypes, however, an increasing balance of M2 macrophages relative to lymphocyte infiltration has been shown to be predictive of shorter survival (16, 20). Among lymphocytes, an increased proportion of CD8 relative to CD4 T cells has been associated with shorter survival (16, 24, 25). However, this effect was found to be reversed following neoadjuvant chemotherapy in one early study (26). Post-treatment immunologic alterations remain an area of active study.

**Table 1:** Summary of key findings.

Findings	Reference(s)
**Myeloid Infiltrate**	
Macrophages and lymphocytes comprise a significant portion of the tumor microenvironment	Burt et al. (15); Losi et al. (25)
Increased macrophage infiltration predicts shorter survival in non-epithelioid MPM, but not in epithelioid MPM	Burt et al. (15) Cornelissen et al. (14)
Non-epithelioid tumors exhibit higher levels of immunosuppressive M2 macrophage-associated gene expression	Bueno et al. (20)
Higher proportion of CD163^+^-M2 macrophages is associated with shorter survival relative to:	
• CD8 T cells (CD163/CD8 ratio)	Ujiie et al. (16)
• CD20 B cells (CD163/CD20 ratio)	Ujiie et al. (16)
• overall T cells (by canonical gene expression profiles)	Bueno et al. (20)
**Lymphoid Infiltrate**	
PD-L1-positive tumors exhibit higher overall T cell infiltration, CD8 memory T cells, CD8^+^ cell proliferation, CD4 T cells, and CD19 lymphocytes	Awad et al. (21) Fusco et al. (24)
Higher PD-L1 expression is associated with poor prognosis	Combaz-Lair et al. (22) Losi et al. (25)
Higher proportion of CD4 T cells is associated with longer survival	Fusco et al. (24); Ujiie et al. (16)
Higher proportion of CD8 T cells is associated with shorter survival	Fusco et al. (24); Losi et al. (25)
Post-neoadjuvant chemotherapy, high CD8 cells paradoxically demonstrate trend toward longer survival.	Anraku et al. (26)

Investigations of the effect of checkpoint molecule expression, including PD-L1, are also ongoing. Studies have shown that higher PD-L1 expression is associated with shorter survival (25). Non-epithelioid tumors exhibit higher PD-L1 expression than epithelioid tumors (23), and there is substantial transcriptomic variability among the expression of checkpoint molecules along the epithelial-to-mesenchymal spectrum observed in a large series of MPM transcriptomes (51).

Genetic alterations in MPM have also been associated with different immune subtypes. *BAP1* mutations have been linked to upregulation of the local innate immune response in peritoneal mesothelioma (42), but no significant association has been shown for pleural mesothelioma in either TCGA-based or other analyses (52). In MPM, aneuploidy is quite common and the creation of novel gene products through chromosomal rearrangements has significant immune implications (27, 38). Epigenetic alterations have been identified, particularly in peripheral DNA specimens in asbestos-exposed MPM cases, and have been associated in limited contexts with altered inflammatory and immune pathways (33). However, further work is needed to assess for causal relationships between these findings and specific changes in the tumor microenvironment. Gene expression is a widely studied approach to predicting the immunologic behavior of a given tumor. Association of both transcriptomic information and IHC with clinical data has revealed distinct patterns of immune activation or suppression (20, 50–52).

There remain several gaps to be addressed to link genetic signatures to the TME, and especially to predict clinical outcomes and guide therapy. Current and future work with high-resolution sequencing technology will identify unique immune programs to associate the genetic characteristics of individual tumor to specific immune phenotypes. Next-generation sequencing and machine learning are being applied to develop polygenic scoring systems predictive of response to checkpoint therapy. Spatial transcriptomics can reveal crosstalk between tumor cells and adjacent leukocytes, as well as interactions between different immune and stromal cells within the microenvironment. Genotypic inferences can be made from single-cell transcriptomic data, allowing correlation between cell clonality and immune behavior in different regions of a given tumor. As improved murine and *in vitro* tumor models are being developed, immunomodulatory therapeutics can be readily tested on patient-specific tissues leading to personalized medicine. Ultimately, full understanding of the association between immune TME and genetics will lead to improved prognostication and outcomes for patients with MPM.

## Author Contributions

Literature review: BW and ADR. All authors contributed to the article and approved the submitted version.

## Conflict of Interest

RB reports research grants and clinical trials support from MedGenome, Roche, Verastem, Genentech, Merck, Gritstone, Epizyme, Siemens, National Institutes of Health, and Department of Defense. In addition, RB holds 4 patents through Brigham and Women’s Hospital and equity in Navigation Sciences.

The remaining authors declare that the research was conducted in the absence of any commercial or financial relationships that could be construed as a potential conflict of interest.
